# Microglia in Multiple Sclerosis: Friend or Foe?

**DOI:** 10.3389/fimmu.2020.00374

**Published:** 2020-03-20

**Authors:** Brooke L. Guerrero, Nancy L. Sicotte

**Affiliations:** Multiple Sclerosis and Neuroimmunology Program, Department of Neurology, Cedars-Sinai Medical Center, Los Angeles, CA, United States

**Keywords:** multiple sclerosis, microglia, pathology, quantitative susceptibility mapping, PET imaging, biomarkers, disease modifying therapy

## Abstract

Microglia originate from myeloid progenitors in the embryonic yolk sac and play an integral role in central nervous system (CNS) development, immune surveillance and repair. The role of microglia in multiple sclerosis (MS) has been complex and controversial, with evidence suggesting that these cells play key roles in both active inflammation and remyelination. Here we will review the most recent histological classification of MS lesions as well as the evidence supporting both inflammatory and reparative functions of these cells. We will also review how microglia may yield new biomarkers for MS activity and serve as a potential target for therapy.

## Introduction: Microglia in Development and Disease States

Microglia populate the CNS during embryonic development and are believed to derive from myeloid precursors in the yolk sac, making them distinct from monocyte derived macrophages (1). They play a crucial role in refining synaptic networks through pruning, developmental apoptosis, positioning of neurons in the barrel cortex, and secretion of growth factors (2–6). Microglia are also now recognized to be sexually dimorphic with implications for diseases that are more common in one gender like autism (7). Mutations in the microglia specific gene Colony Stimulating Factor 1 Receptor (CSF1R) have been shown to underlie the newly defined diseases pediatric onset leukoencephalopathy with congenital absence of microglia and adult onset leukoencephalopathy with axonal spheroids and pigmented glia, further illustrating the integral nature of these cells to the normal development and maintenance of the CNS (8, 9).

Microglia in the homeostatic state were classically designated by a distinct morphology characterized by delicate branches, previously referred to as “resting state” (1). However, this term is no longer favored since these cells actively survey the CNS environment and quickly respond to signs of neuronal distress (10). When “activated” during pathological states, microglial morphology changes to resemble the typical amoeboid appearance of a macrophage and yet, morphology alone does not accurately reflect activation (1, 11). Various cell surface markers have been explored in order to differentiate microglia from macrophages and identify microglia in the homeostatic state (Table 1). Cell surface markers including transmembrane protein 119 (TMEM119) and purinergic receptor P2Y12 are emerging as more reliable markers of microglial state under pathological conditions (12, 13). The previously favored dichotomy of proinflammatory (M1) and anti-inflammatory (M2) microglia is no longer considered valid since evidence now indicates that microglial phenotypes are transient and demonstrate temporal and spatial evolution (1, 11, 14). An intriguing new phenotype, deemed “dark microglia,” has also been discovered that may play a role in pathological remodeling of neuronal circuits (15). Variations in genetic expression within CNS tissue types adds another layer of complexity in assessing microglial activity in inflammatory disorders. For example, new data indicates that there is higher expression of type I interferon and complement genes in gray matter and higher expression of NF-κB inhibitor genes in white matter (16).

**Table 1:** Markers indicating microglial state.

**Microglial state**	**Marker**
Non-specific (also present on macrophages)	CD68 (transmembrane glycoprotein scavenger receptor) Iba1 (calcium binding adapter molecule-1)
Homeostatic	P2RY12 (purinergic (ADP, ATP) G protein coupled receptor) TMEM119 (transmembrane protein) CX3CR1 (fractalkine receptor)
Pro-inflammatory/Disease associated	P22phox (NADPH oxidase) CD86 (co-stimulatory T cell signal) MHC class II antigens Ferritin (iron storage) Trem2 (triggering receptor on myeloid cells, promotes phagocytosis)
Anti-inflammatory/Pro-regenerative	CD206 (mannose receptor) CD163 (haptoglobin-hemoglobin scavenger receptor) Arginase-1 IGF-1(Insulin like growth factor-1) TGF-beta (transforming growth factor-beta)

Aside from the genetic diseases mentioned above, microglia have increasingly been implicated in neurodegenerative diseases, including Alzheimer disease, Parkinson disease, amyotrophic lateral sclerosis, and multiple sclerosis (17–21). Within the context of MS, “classically activated” microglia are thought to be critical for phagocytosis of myelin, antigen presentation to T cells and release of proinflammatory cytokines in active lesions (22). In experimental autoimmune encephalomyelitis (EAE) models, microglial paralysis has been shown to both delay EAE onset and reduce clinical severity (23). In addition to the role microglia play in inflammatory lesion formation, they are equally crucial for clearing myelin debris and enabling remyelination which reflects a change to an “alternatively activated” or anti-inflammatory state (24). And yet as MS shifts into the progressive phase, microglia are again implicated in the slow expansion of chronic lesions. These lesions, detectable on phase contrast imaging, are thought to result from a complex compartmentalized inflammatory process behind an intact blood brain barrier (22). However, these lesions have not been routinely assessed in clinical trials and have not been targeted for treatment as of yet.

Increasingly, the role of microglia is recognized as a key player in not only MS pathology but multiple inflammatory and degenerative diseases. A better understanding of the complex activities of these cells and identifying ways to either target or harness their activity is likely to have application across a wide spectrum of neurodegenerative disorders.

### Histological Classification of MS Lesions

Active MS lesions, typically found in early relapsing remitting MS (RRMS), are characterized by diffuse infiltration with microglia, peripheral macrophages, T lymphocytes and plasma cells (25, 26). These lesions can be either demyelinating or post-demyelinating depending on the presence of intracytoplasmic myelin breakdown products (25). Early demyelinating lesions contain microglia/macrophages with both minor myelin proteins (MOG, CNP and MAG) as well as major myelin proteins (MBP and PLP) (25). Late demyelinating lesions demonstrate only major myelin proteins (25). Active lesions are heterogenous and can be subdivided into four distinct patterns (pattern I, II, III, and IV) based on criteria first described by Lucchinetti et al. (26). Pattern I is the “standard” active lesion with the basic features mentioned above. Pattern II lesions are distinguished by evidence of immunoglobulin and complement deposition. Pattern III lesions show a selective loss of MAG and oligodendrocyte apoptosis. Pattern IV lesions demonstrate non-apoptotic loss of oligodendrocytes and were only observed in primary progressive MS (PPMS) patients in the original study (26). Cortical demyelinating lesions, which can be subdivided into leukocortical, subpial, and intracortical lesions, were first described in secondary progressive MS (SPMS) and PPMS but are now known to also be a feature of the very earliest stages of MS (27, 28). Lesions with evidence of remyelination, also known as “shadow plaques,” are distinguished by the presence of thin myelin sheaths and are more common alongside active lesions. Tumefactive MS lesions mostly resemble typical active MS lesions but can have Creutzfeldt cells that can be misinterpreted as mitotic figures but actually represent reactive astrocytes with fragmented nuclear inclusions (29). Tumefactive lesions are largely overrepresented in post-mortem pathology studies in MS since it is usually the tumefactive appearance of lesions that prompts either biopsy or autopsy.

Mixed active/inactive lesions, also termed “smoldering,” “slowly expanding,” or “chronic” are defined by a hypocellular lesion center surrounded by a rim of activated macrophages/microglia (25, 30). A higher proportion of this type of lesion, along with total lesion load, correlate with greater severity of disease (31). Inactive lesions have few microglia, loss of mature oligodendrocytes and begin to show evidence of axonal loss. These lesions predominate in patients with a long disease duration or non-active SPMS. The criteria for lesion types in MS is summarized in Tables 2, 3.

**Table 2:** Criteria for lesion activity.

**Lesion type**	**Histological features**
Active	***Pattern I***: activated microglia/macrophages (CD68), T cells (CD3), centered around veins/venules
	***Pattern II***: activated microglia/macrophages, T cells, immunoglobulin (IgG) and complement (C9neo), centered around veins/venules
	***Pattern III***: activated microglia/macrophages, T cells, ill-defined borders, not centered around veins/venules, selective loss of MAG, oligodendrocyte nuclear condensation/fragmentation (apoptotic), no remyelinated lesions
	***Pattern IV***: activated microglia/macrophages, T cells, non-apoptotic oligodendrocyte degeneration in the periplaque white matter adjacent to an active lesion, no remyelinated lesions
Mixed active/Inactive	Hypocellular lesion center with rim of activated macrophages/microglia
Inactive	Sharply demarcated, hypocellular, few mature oligodendrocytes, loss of axons

**Table 3:** Criteria for evidence of demyelination.

**Demyelinating**	**Post-demyelinating**	**Re-myelinated**
***Early***: positive for MOG, CNP, MAG, MBP and PLP, phagocytes positive for MRP14 ***Late***: positive for MBP, PLP	Positive only for PAS (non-specific debris)	Thin myelin sheaths in a sharply demarcated plaque

*MBP, Myelin basic protein, MOG, Myelin oligodendrocyte glycoprotein, PLP, Proteolipid protein, MAG, myelin associated glycoprotein, CNP, cyclic nucleotide phosphodiesterase*.

The two main differential diagnoses for demyelinating lesions are acute disseminated encephalomyelitis (ADEM) and neuromyelitis optica spectrum disorder (NMOSD). ADEM has cortical microglial aggregates but unlike MS, they are not associated with cortical demyelination, and the inflammatory infiltrate includes macrophages, lymphocytes, and granulocytes (32). NMOSD pathology is dominated by loss of aquaporin 4 immunoreactivity, loss of astrocyte markers such as glial fibrillary associated protein and a vasculocentric deposition of IgG, IgM, and terminal complement components (29). Microglial infiltration and lipid laden macrophages are detected in NMOSD but smoldering lesions with activated microglial rims do not appear to be a feature of this antibody mediated inflammatory disorder which is a disease of relapses and does not have a progressive phase like MS (29).

### Microglia and MS Pathogenesis

The initial pool of phagocytic cells in an early MS lesion is comprised of roughly 40% microglia as measured by the marker TMEM119, which is expressed exclusively on microglia and not on macrophages (13, 33). Peripheral macrophages are increasingly recruited as the lesion progresses (33). Virtually none of the microglia in an active lesion are homeostatic, as determined by the presence of P2RY12, an ADP receptor that is specific for the ramified processes of microglia seen in the resting state (33–35). Even in the normal appearing white matter of MS patients there are nodules of activated microglia, but whether these microglia are homeostatic or activated is debatable since one study showed a loss of P2RY12 but another showed unaltered P2RY12 gene expression (16, 33). The significance of these nodules of activated microglia remains unclear since they either represent the earliest stage of MS or the by-product of Wallerian degeneration from an upstream lesion (14). The regional heterogeneity of microglia, which had originally been reported in mice, was also found to have a disease specific manifestation in progressive MS patients who demonstrate an upregulation of genes involved in lipid processing in normal appearing white matter and iron homeostasis in normal appearing gray matter (16, 36). This demonstrates that metabolic changes in microglia that mirror MS pathology are detectable in the absence of demyelinating lesions and highlight the differential inflammatory processes seen in white and gray matter in MS.

Active demyelination is usually associated with a pro-inflammatory microglia phenotype (positive for p22phox, CD68, CD86, and Class II MHC antigens) while anti-inflammatory markers (CD206, CD163, ferritin) peak in the inactive lesion center (33, 37). In multiple animal models, internalization of myelin by microglia leads to a pro-regenerative phenotype expressing arginase-1, CD206, and insulin-like growth factor-1 (IGF-1) which facilitates oligodendrocyte differentiation and is necessary for remyelination (34, 38–41). Evidence in humans points to the involvement of microglia in balancing bone morphogenetic protein 4, which may impede remyelination, and its antagonist Noggin, which is more highly expressed in remyelinated lesion areas (42).

Interestingly, microglia in the normal human brain have an intermediate activation state (reduced P2RY12 and the presence of CD68) which is different than the homeostatic state found in animal models. These findings suggest that microglia in humans may differ from other species perhaps due to higher levels of systemic inflammation at time of autopsy (14, 33). In the MS brain, microglia from normal appearing white matter were found to be unresponsive to lipopolysaccharide (LPS) and had other evidence of diminished inflammatory responsiveness, despite their activated phenotype (43).

MS susceptibility genes were recently found to be more frequently associated with microglia function than neurons or astrocytes (44). These intriguing findings place microglia at the center of MS pathogenesis. Mutations in CSF1R, a key microglial specific gene which is associated with other leukoencephalopathies, has not been associated with MS pathology and sequencing of CSF1R in MS patients did not identify any relevant mutations (45).

### Neuroimaging Methods To Detect Microglial Activation

Some progress has been made in developing neuroimaging approaches to corroborate microglial activity seen in animal models and post mortem tissues. These developments are key to improving the ability to quantify microglial activation *in vivo*, assess longitudinal changes and determine how to monitor responses to disease modifying therapy.

Translocator Protein (TSPO) is located in the outer mitochondrial membrane and is upregulated in activated microglia (46, 47). Positron emission tomography (PET) imaging with radiotracers that target TSPO have been used in humans. The tracer 11C-(R)-PK11195 has shown high binding of TSPO in acute MS lesions as well as the normal appearing white matter of clinically isolated syndrome (CIS), RRMS and SPMS (46, 48–52). This binding decreases in both acute lesions and normal appearing white matter after treatment with highly effective therapies such as natalizumab (48, 49). Whole brain 11C-(R)-PK11195 binding potential also decreased after 1 year of treatment with glatiramer acetate in both cortical gray matter and cerebral white matter (53). Fingolimod reduced 11C-(R)-PK11195 binding within the combined T2 lesion area after 6 months of treatment but not in the areas of normal appearing white matter or gray matter (54). Higher binding in the normal appearing white matter has been shown to be more common in SPMS compared to RRMS and associated with greater white matter disruption as measured by lower fractional anisotropy and higher clinical disability (55). The binding potential of 11C-(R)-PK11195 surrounding T1 black holes was found to correlate with EDSS in progressive patients but not relapsing patients (50). A different tracer, [11C]DPA713, showed persistent elevation in the cortex and normal appearing white matter of MS patients despite DMT (56). Baseline distribution volume ratio in the normal appearing white matter using the radioligand 11C-PBR28 was correlated with enlarging T2-hyperintense lesion volumes in RRMS patients and brain atrophy in SPMS patients (57).

The other method employed to monitor microglia is quantitative susceptibility mapping (QSM) assessed with MRI. QSM detects high tissue susceptibility at the rims of MS lesions that correlates with the distribution of iron positive microglia (58). The iron rims detected by QSM are thought to represent slowly expanding lesions related to pro-inflammatory microglia as seen on post mortem tissue. However, QSM can lack specificity, as areas of high susceptibility have also been attributed to myelin loss (58). *In vivo* studies have shown that lesions with rims show significant expansion over time compared to lesions without rims (59). Patients with active RRMS have more lesions with rims than patients with stable disease (60, 61). Rim lesions can persist for years and are associated with higher conversion to T1 black holes (62).

The interaction between QSM and TSPO was explored in a study that found that 11C-(R)-PK11195 uptake was higher in rim positive lesions compared to rim negative lesions and this was also confirmed with post mortem immunohistochemistry for iron containing CD68 positive cells (63). These findings suggest that QSM detectable rims do contain activated microglia. The major factors limiting the use of TSPO PET for routine clinical testing include patient exposure to radioactivity, genetic polymorphisms that affect binding of the tracer, and potential lack of specificity because peripheral macrophages and astrocytes can also upregulate TSPO (64, 65). In addition, TSPO expression in human microglia has also been reported to be reduced in response to pro-inflammatory stimulation with LPS and interferon gamma, which is the exact opposite of the increased expression seen in mouse microglia, raising concerns about the specificity of TSPO as a marker of microglial activation (66).

Other new intriguing PET radiotracers that have currently only been studied in animal models include P2X7 which is a trimeric ATP-gated cation channel found predominantly, but not exclusively, on microglia, P2RY12, and sphingosine 1 phosphate receptor (SIPR) (67). P2X7 is thought to be associated with proinflammatory microglia and is upregulated during pathological states. The P2X7 antagonist, GSK1482160, radiolabeled with carbon-11 showed increased accumulation in the brains of LPS treated mice and in the lumbar spinal cord of EAE mice suggesting it is a sensitive marker of inflammation (67). P2RY12 would be a good marker of homeostatic microglia but so far the only tracer studied, 11C-2, showed only *in vitro* binding in the mouse brain and rapid plasma metabolism making it less attractive for use in humans (67). S1P receptors are the target of the MS disease modifying therapy fingolimod, whose therapeutic benefit is thought to be due to peripheral effects on circulating lymphocytes. But S1P receptors are also found on activated microglia and the use of 11C-TZ3321, an S1P receptor antagonist, showed higher uptake in the lumbar spinal cord of EAE rats, making it an interesting target to monitor inflammation (67).

### Emerging Microglia Biomarkers

Given the limited specificity and clinical limitations of imaging modalities to detect and monitor microglial activity, other approaches are being developed to serve as better biomarkers. Proteomics is one approach that may offer more informative biomarkers of microglial activity in body fluids with the added the ability to assess cell specific processes in living patients. New advances in “cell specific” proteomics have been developed and tested in MS that provide markers of the cell of origin, greatly increasing the utility of these measures (68). Using a predetermined multiplex proteomic scan, CSF of MS patients yielded elevated astrocytic and microglial markers which were correlated with disease severity as measured by two clinical metrics (Age Related MS Severity and MS Disease Severity Scale). These approaches await validation on a larger scale but offer an attractive option for disease monitoring and discovery science (68).

In a similar vein, the proteomics of extracellular vesicles (EVs) are also being explored as a source for MS biomarkers. EVs are lipid bilayer particles naturally released from cells and although previously thought to be solely a method for protein, lipid and RNA elimination they are now also considered a means of intercellular communication (69). Elevated levels of EVs have been found in MS patients compared to healthy controls originating from various types of cells including monocytes, lymphocytes, and endothelial cells (70). Microglia-derived EVs were recently found to be present in tears, mirroring their levels in CSF (71). Given that a separate analysis of activated genes in the CSF and tears of MS patients revealed activation of TGFB1, the study authors hypothesize that extracellular vesicles may be able to communicate nuclear information and insert it into target cells (71). It would be a great advance to have a readily accessible biofluid such as tears that carried so much information about molecular cross-talk but further validation of these methods is needed prior to clinical implementation.

Lastly, soluble CD163, which is a receptor for haptoglobin-hemoglobin complexes, is secreted in the serum by monocytes but in the CNS likely arises from both macrophages and microglia (72). When incorporated into a panel alongside established MS biomarkers (CXCL13 ratio, neopterin ratio, CSF level of neurofilament light polypeptide, IgG index, and serum level of osteopontin) it improved the diagnostic specificity for differentiating MS patients from symptomatic controls and revealed unique profiles for each subtype of MS (72).

### Microglia as Therapeutic Targets

Current disease modifying therapies (DMT) have been shown to have a modest effect on microglia and are divided into indirect and direct effects (73, 74). Interferon beta and glatiramer acetate exert an indirect effect by inducing a Th2 shift in lymphocyte profile thereby reducing the pro-inflammatory phenotype of microglia (73, 74). Interferon beta suppresses interferon gamma induced MHC class II expression on microglia but paradoxically increases the production of inflammatory mediators such as TNF-α, IL-1β, IL-6, and NO (75, 76). Glatiramer acetate reactive T cells isolated from treated MS patients promoted an alternatively activated phenotype in microglia through indirect effects (77). *In vitro* studies of dimethyl fumarate show inhibition of LPS-induced activation of microglial cells by reducing the expression of TNF-α, IL-1β, IL-6, and NO, likely through activation of the Nrf2 pathway (78). Teriflunomide exerts an indirect effect on microglia through its primary action on lymphocytes but *in vitro* studies indicate that it may reduce microglial proliferation without modulating the microglial phenotype (79). Fingolimod probably has the most direct effect of any DMT given that it can access the CNS and binds directly to S1P receptors on microglia, also leading to downregulation of TNF-α, IL-1β, and IL-6 (80). There is also evidence that fingolimod may augment microglial related remyelination (81). Natalizumab does not appear to have any clear effect on microglia, except as evidenced by the prior discussed TSPO studies, but alemtuzumab seems to indirectly affect microglia through increased production of brain-derived neurotrophic factor, platelet derived growth factor and ciliary neurotrophic factor from reconstituting lymphocytes (82). There are no documented studies regarding the effect of B-cell depleting agents, such as rituximab or ocrelizumab on microglia. In summary, many of the currently available DMTs have effects on microglia by decreasing inflammatory tone. Interestingly, these mostly anti-inflammatory therapies have limited if any, effects on remyelination or progressive disease.

Minocycline is an antibiotic that is currently not approved for use as a DMT in MS. However, there is significant preclinical evidence that minocycline impairs microglial activation thereby reducing the severity of disease in the EAE model (34). Reflecting the contrasting effects of microglia in CNS inflammation, decreased microglial activation may also contribute to a reduced remyelination potential by oligodendrocytes in this setting (39). In clinical trials, minocycline reduced the rate of conversion from clinically isolated syndrome to MS, and demonstrated a benefit on multiple efficacy endpoints including annualized relapse rate when added to glatiramer, but had no effect when added to interferon beta (83–85).

Emerging therapies targeting microglia directly are now being be investigated in the EAE model with promising results. PLX5622 is an oral CSF1R antagonist that inhibits its kinase activity and was shown to preferentially deplete microglia of the M1 phenotype, reduce demyelination, preserve mature oligodendrocytes, and improve mobility in EAE mice (86). Ethyl pyruvate is a redox analog of dimethyl fumarate and was shown to reduce Iba1+ microglia within the CNS and protect against EAE (87). A peptide vaccine therapy (PADRE-Kv1.3) that targets potassium channels on T cells was tested in EAE and led to reduced levels of IL-17, IFN-γ, and IL-1β, decreased numbers of infiltrating microglia, and promoted a shift in the phenotype of microglia from pro-inflammatory (expressing iNOs) to anti-inflammatory (expressing Arginase-1) (88).

## Conclusion

**Figure 1:**
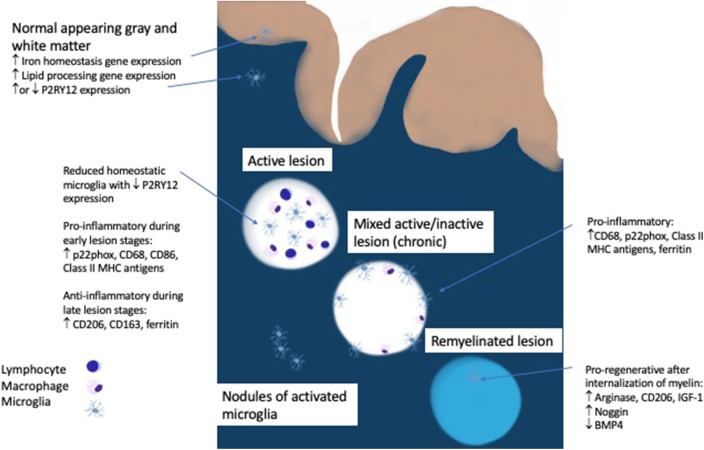
Role of microgila in MS pathology.

Microglia play complex roles in multiple sclerosis related disease activity. They are present throughout all stages of lesion formation as a driver of inflammation, they are detectable in slowly expanding lesions linked to disease progression and they are present diffusely throughout the cortex and contribute to synaptic loss (Figure 1). In contrast, microglia also play important roles in remyelination and in limiting inflammatory responses. The previous classification of M1/M2 or “good” or “bad” microglia fail to capture the complexity and subtly of microglial activity which changes rapidly in response to local conditions as well as tissue type. It is intriguing that many of the newly discovered MS risk genes are highly expressed in microglia. Fully elucidating the downstream effects on microglial function may help to shed light on their role in modulating or exacerbating inflammatory activity in the CNS. Emerging biomarkers should help to track the activity of these vital cells and lead us closer to more targeted therapies, not only in MS but in other neurodegenerative disorders such as Alzheimer and Parkinson Disease in which microglia have been implicated. Ultimately, these approaches will need to maintain the delicate balance of all aspects of microglial function in preserving brain homeostasis and health.

## Author Contributions

The manuscript concept was developed by BG and NS. BG researched and wrote the manuscript. NS provided guidance, reviewed, and edited the manuscript.

### Conflict of Interest

The authors declare that the research was conducted in the absence of any commercial or financial relationships that could be construed as a potential conflict of interest.
